# Pattern recognition as a concept for multiple-choice questions in a national licensing exam

**DOI:** 10.1186/1472-6920-14-232

**Published:** 2014-11-14

**Authors:** Tilo Freiwald, Madjid Salimi, Ehsan Khaljani, Sigrid Harendza

**Affiliations:** Department of Nephrology, III. Medical Clinic, Goethe-University Hospital, Theodor-Stern-Kai 7, 60590 Frankfurt/Main, Germany; MIAMED GmbH, Sachsenring 73, 50677 Köln, Germany; Department of Urology, Vivantes Auguste-Viktoria-Clinic, Rubensstraße 125, 12157 Berlin, Germany; Department of Internal Medicine, University Hospital Hamburg-Eppendorf, Martinistr. 52, 20246 Hamburg, Germany

**Keywords:** Multiple-choice questions, Pattern recognition, Clinical reasoning, eLearning

## Abstract

**Background:**

Multiple-choice questions (MCQ) are still widely used in high stakes medical exams. We wanted to examine whether and to what extent a national licensing exam uses the concept of pattern recognition to test applied clinical knowledge.

**Methods:**

We categorized all 4,134 German National medical licensing exam questions between October 2006 and October 2012 by discipline, year, and type. We analyzed questions from the four largest disciplines: internal medicine (n = 931), neurology (n = 305), pediatrics (n = 281), and surgery (n = 233), with respect to the following question types: knowledge questions (KQ), pattern recognition questions (PRQ), inverse PRQ (IPRQ), and pseudo PRQ (PPRQ).

**Results:**

A total 51.1% of all questions were of a higher taxonomical order (PRQ and IPRQ) with a significant decrease in the percentage of these questions (p <0.001) from 2006 (61.5%) to 2012 (41.6%). The proportion of PRQs and IPRQs was significantly lower (p <0.001) in internal medicine and surgery, compared to neurology and pediatrics. PRQs were mostly used in questions about diagnoses (71.7%). A significantly higher (p <0.05) percentage of PR/therapy questions was found for internal medicine compared with neurology and pediatrics.

**Conclusion:**

The concept of pattern recognition is used with different priorities and to various extents by the different disciplines in a high stakes exam to test applied clinical knowledge. Being aware of this concept may aid in the design and balance of MCQs in an exam with respect to testing clinical reasoning as a desired skill at the threshold of postgraduate medical education.

## Background

Multiple-choice questions (MCQs) are still used in high stakes exams worldwide to assess the knowledge of medical students. Even though alternative assessment formats are available and increasingly applied, such as modified essay questions (MEQs) or objective structured clinical exams (OSCEs), the ease of use and testing efficiency of these formats are tempting features for the continued and widespread application of MCQs. In the USA and Germany, for example, MCQs constitute a major part of the National Medical Licensing Exam. While MCQs were originally designed to assess factual knowledge, well-constructed MCQs can also assess the application of knowledge, resembling a taxonomically higher order than the simple recall of isolated facts [1]. Answering ‘higher order’ MCQs still requires cognitive knowledge, yet their realism receives greater acceptance by students and teachers [2, 3]. Cognitive knowledge alone does not guarantee competence, which integrates knowledge, skills, and attitudes [4]. However, Glaser has already demonstrated in a developmental study in 1984, that knowledge is the single best determinant of expertise [5]. This raises the question of whether ‘higher order’ MCQs might provide an opportunity to test the clinical reasoning skills of medical students.

Clinical reasoning used by physicians in daily practice presents itself as a combination of two different approaches: diagnostic pattern recognition (PR) and analytical hypothesis-based thinking [6]. The ability of students to succeed in PR and clinical data interpretation shows a steady growth curve over increasing years at medical school [7, 8]. In a study to determine the relationship between problem-solving strategies and the likelihood of diagnostic success, the latter was significantly greater when study participants used PR rather than hypothetico-deductive, i.e. analytical, reasoning [9]. While PR appears to happen unconsciously and almost automatically, the process of making an instant diagnosis is still based on the recognition of distinctive features of a certain disease and is a reasoning strategy widely used by medical experts with many years of experience [10]. Even though PR is an important diagnostic tool and should be taught as a clinical reasoning strategy at medical school, clinicians must be aware that patterns can become rigid and the excessive focus on favorite patterns can lead to diagnostic errors when key features are prematurely assumed to represent a particular disease [11]. Novices or unreflective physicians might focus too much on looking for the presence of specific patterns and may overlook other potentially important information [12]. Nonetheless, medical students need to be familiar with PR and clinical data interpretation as diagnostic reasoning strategies and need to familiarize themselves with both of these principles during their undergraduate studies. However, analytical thinking requires feedback [13] and, therefore, cannot be applied in MCQ exams in a similar way.

Even though PR is, by definition, a personal and idiosyncratic process and might not be explicitly taught as clinical reasoning in every medical school, we hypothesize that it is an ideal concept to test applied medical knowledge in high stakes exams. To test whether and to what extent PR is used in MCQs, we defined a framework for the detection of disease patterns clinically used for PR. Based on this framework we analyzed all MCQs from the German National Licensing Exams, Part 2, between October 2006 and October 2012 in the disciplines of internal medicine, surgery, neurology, and pediatrics.

## Methods

Since October 2006, every German National Medical Licensing Exam, Part 2, has consisted of 320 MCQs and an additional oral-practical exam lasting two hours per student. The exam takes place at the end of the final year of a six-year medical undergraduate curriculum and is held twice a year, in April and October. The actual final number of valid questions per exam is often below 320, because invalid MCQs are excluded after the exam has taken place. All MCQs have five possible answers and include questions with either a single correct or a single incorrect answer. In addition, long patient cases with six to 17 questions related to the same case are presented, with a single correct answer per question. Extended-matching MCQs are not included in this exam.

MCQs for the German National Medical Licensing Exam, Part 2, are developed by a national institute (IMPP, Institute for medical and pharmaceutical national exam questions). Panelists, recruited from the different specialist medical societies develop and revise the questions with respect to their scientific and clinical content. In a second step, IMPP employees check the questions with respect to formal correctness, comprehensibility, and difficulty. In a third step, referees in different panels solve the anonymized questions and discuss and revise them afterwards, if necessary, with respect to content and structure. The vote to actually use a certain question in an exam has to be unanimous.

We screened a total of 4,134 questions from the German National Medical Licensing Exam, Part 2 (October 2006 until October 2012), and assigned each question to one of 23 medical disciplines based on its topic and the correct answer. Questions from the four largest disciplines (internal medicine, surgery, neurology, and pediatrics), which constituted more than 42% of all questions, were included in this study. Questions from other disciplines, such as ophthalmology, were excluded from our analysis, because their numbers per discipline were too small for statistical analysis. In certain years, some disciplines were not even included in the exam. Questions were assigned to the pediatric discipline when the age of the described patient was below 18 years. When an overlap between internal medicine and surgery was detected, questions were assigned to the surgery discipline when surgical procedures were the correct treatment. This resulted in 1,750 questions, which were included in our analysis (internal medicine: n = 931, neurology: n = 305, pediatrics: n = 281, surgery n = 233).

We defined four categories of questions for our analysis: PR questions (PRQ), pseudo PR questions (PPRQ), inverse PR questions (IPRQ), and knowledge questions (KQ). An example of every type of question is given in Table 1. PRQs include questions describing a patient case or a typical disease pattern, using either one symptom or a combination of symptoms, results of laboratory and other tests, and other information from a patient’s history that is relevant to a medical pattern. Related to the types of answers, these questions were subcategorized into PRQ/diagnosis, PRQ/diagnostic procedures, PRQ/pathophysiology, and PRQ/therapy. KQs ask for mere facts in connection with a disease or symptom, resembling K1 (“recall and comprehension”), as described by Ware and Vik [14]. In a modified Bloom’s taxonomy suggested by Palmer and Devitt, KQs resemble level I (“recall of information”) [15]. IPRQs ask for symptoms or signs that are part of a disease pattern. Signs can also include laboratory findings, for instance. Therefore, to select or combine disease patterns in IPRQs, interpretation of data and/or pathophysiological relationships may be required to find the correct answers. We considered PRQ and IPRQ as questions of a taxonomically higher order, resembling K2 (“application and reasoning”), as described by Ware and Vik [14], or resembling level II (“understanding and being able to interpret data”), and occasionally level III (“use of knowledge and understanding in new circumstances”) by Palmer and Devitt [15]. PPRQs describe a patient case, but mention the diagnosis at the end of the text, which we considered a pitfall in the construction of a potential PRQ, because it is not necessary to read the case in order to answer the question. This downgrades a potential PRQ to a mere KQ.

**Table 1:** **Examples for the different types of MCQs**

PR question (PRQ)	A 26-year-old man presents with increased thirst, urinary frequency and nocturia over the past several months. Physical examination is unremarkable. Twenty-four-hour urine osmolarity is <300 mOsm/L. A fluid deprivation test does not result in an increased urine osmolarity. Administration of 0.03 μg/kg of desmopressin results in a urine osmolarity of 450 mOsm/L after 2 hours. Which of the following is the most likely diagnosis?
Pseudo PR question (PPRQ)	A 42-year-old female consults her general practitioner because of increasing frequency of diarrhea with voluminous stools. The symptoms started six months ago and she is moving her bowels up to five times a day. Furthermore, she complains of flatulence and loss of weight (3 kg). Further investigations result in the diagnosis of celiac disease. Which diagnostic finding confirms this diagnosis?
Inverse PR question (IPRQ)	Different symptoms can lead to the diagnosis of renal artery stenosis; which symptom does not belong in this list?
Knowledge question (KQ)	Which type of bleeding is typical for low platelets?

Each question was assessed and categorized by two of four physician panelists. When disagreement in categorizing occurred, the question was discussed with one of the other two panelists and categorized according to the best fitting category according to the descriptions mentioned above.

Data were analyzed using SPSS statistical software (version 21). We assessed differences between the question categories and between the different disciplines with the *χ*^2^-test and significance levels of p <0.05.

## Results

**Figure 1:**
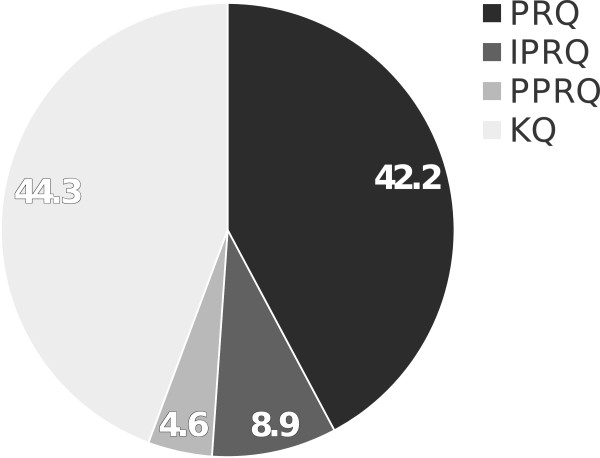
**Overall distribution of question categories between 2006 and 2012.** Disciplines of surgery, pediatrics, neurology, and internal medicine are combined. Numbers resemble %.

**Figure 2:**
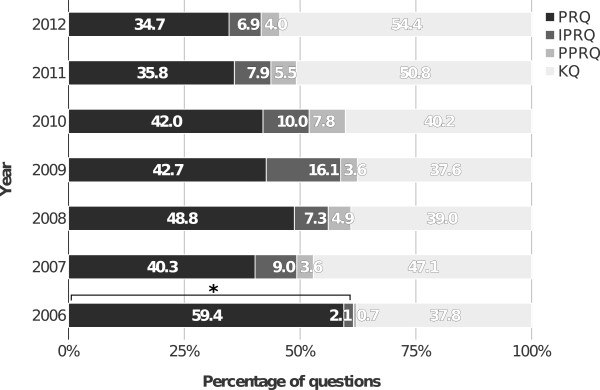
**Distribution of question categories per individual year.** Disciplines of surgery, pediatrics, neurology, and internal medicine are combined. *p <0.001 (2006 versus 2012).

KQs were identified to constitute the single largest question category (44.3%), while 51.1% of all questions were of a higher taxonomy (PRQ 42.2% and IPRQ 8.9%), and PPRQs occurred in only 4.6% (Figure 1). When we compared the distribution of question categories across the years 2006 to 2012, a significant (p <0.001) decrease of higher order taxonomy questions was noticeable, from 61.5% in 2006 to 41.6% in 2012 (Figure 2).

**Figure 3:**
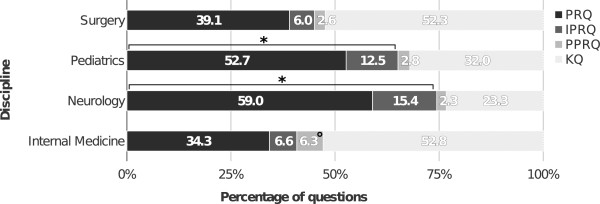
**Overall distribution of question categories per discipline.** *p <0.001 (questions of a taxonomically higher order, i.e. PRQ + IPRQ: internal medicine or surgery versus neurology or pediatrics, respectively), °p <0.05 (PPRQ from internal medicine versus neurology, pediatrics, or surgery).

Analysis of the question categories per discipline revealed that the proportion of higher order taxonomy questions was significantly lower (p <0.001) in internal medicine (40.9%) and surgery (45.1%), when compared with either neurology (74.4%) or pediatrics (65.2%) (Figure 3). The percentage of PPRQs was significantly higher (p <0.05) in internal medicine (6.3%) than in neurology (2.3%), pediatrics (2.8%), or surgery (2.6%). The development of question types per discipline over the years is shown in Table 2.

**Figure 4:**
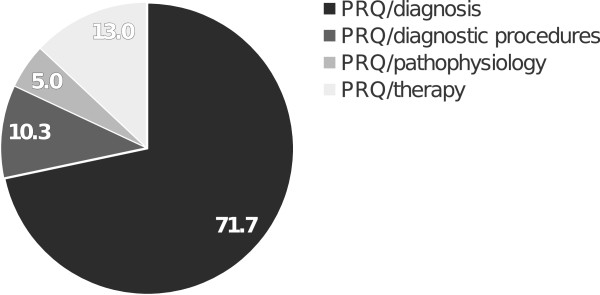
**Overall distribution of subgroups of PR questions in the combined disciplines.** Disciplines: surgery, pediatrics, neurology, and internal medicine. Numbers resemble %.

**Figure 5:**
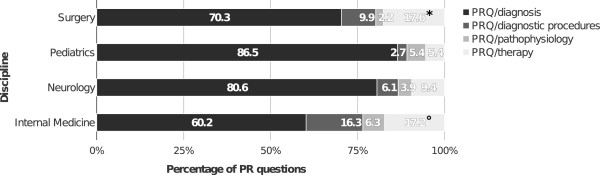
**Distribution of subgroups of PR questions per discipline.** *p <0.05 (PRQ/therapy surgery versus pediatrics). °p <0.05 (PRQ/therapy internal medicine versus neurology or pediatrics).

Subgroups of the PR questions are displayed in Figure 4. The largest subgroup was composed of PR/diagnosis questions (71.7%), while PR/therapy questions occurred in 13.0%, PR/diagnostic procedures questions in 10.3%, and PR/pathophysiology questions in 5.0%. The analysis of PRQs in the different disciplines revealed a significantly higher (p <0.05) percentage of PRQ/therapy questions for internal medicine (17.2%), as compared with neurology (9.4%) or pediatrics (5.4%), as well as for surgery (17.6%) compared with pediatrics (Figure 5). Pediatrics comprised the lowest percentage of PRQ/diagnostic procedures questions (2.7%) compared to any of the other disciplines (internal medicine: 16.3%, surgery: 9.9%, neurology: 6.1%).

**Table 2:** **Question types per discipline and year**

		Question type				
Discipline	Year	PRQ (%)	IPRQ (%)	PPRQ (%)	KQ (%)	Sum (%)
**Surgery**	**2012**	53.6	7.1	3.6	35.7	100
	**2011**	30.6	5.6	2.8	61.0	100
	**2010**	40.5	8.1	0.0	51.4	100
	**2009**	43.3	1.,0	3.3	43.4	100
	**2008**	17.4	8.7	4.3	69.6	100
	**2007**	28.6	0.0	2.9	68.5	100
	**2006**	90.5	0.0	0.0	9.5	100
**Pediatrics**	**2012**	58.9	8.8	2.9	29.4	100
	**2011**	48.2	1.,6	3.6	28.6	100
	**2010**	41.5	1.,9	3.8	35.8	100
	**2009**	52.5	1.5	5.0	30.0	100
	**2008**	65.7	8.6	0.0	25.7	100
	**2007**	62.8	7.0	2.3	27.9	100
	**2006**	40.0	0.0	0.0	60.0	100
**Neurology**	**2012**	48.0	1.0	2.0	34.0	100
	**2011**	50.0	18.8	3.1	28.1	100
	**2010**	69.2	15.4	0.0	15.4	100
	**2009**	60.0	22.0	2.0	16.0	100
	**2008**	61.5	9.6	5.8	23.1	100
	**2007**	49.0	19.1	2.1	29.8	100
	**2006**	80.0	5.7	0.0	14.3	100
**Internal Medicine**	**2012**	22.2	3.7	4.9	69.2	100
	**2011**	28.5	0.8	7.7	63.0	100
	**2010**	35.5	5.9	13.2	45.4	100
	**2009**	34.4	16.2	3.9	45.5	100
	**2008**	50.4	5.3	6.2	38.1	100
	**2007**	34.0	8.5	4.6	52.9	100
	**2006**	44.8	1.5	1.5	52.2	100

## Discussion

The dual process theory of reasoning includes a fast and intuitive approach and a slow and analytical approach [16]. Experts tend to use the intuitive approach more often than novices do; however, when they cannot refer to the pattern of an illness script (a collection of signs and symptoms) [17], they use hypothetico-deductive reasoning as an analytical approach [18]. Since pattern recognition is the fast approach of clinical reasoning applied every day by physicians, we hypothesized that it would occur in high stakes MCQ exams as a relevant concept. We identified 51.1% of all questions from internal medicine, surgery, neurology, and pediatrics from the German National Licensing Exam between 2006 and 2012 to be taxonomically higher order questions involving pattern recognition. However, their proportion dropped continuously from 61.5% in 2006 to 41.6% in 2012, which was way below the suggested level of at least 50% taxonomically higher order questions in MCQ exams [14]. We also detected almost 5% of PPRQs as being ill defined PRQs, which resemble KQs, a pitfall in question design that could be added to a suggested list of common MCQ pitfalls [19]. This can easily be avoided when panelist involved in designing questions are aware of it and will improve the quality of PRQs, raising the overall number of taxonomically higher order MCQs in an exam. However, item writing flaws are still a problem in high stakes exams, as has been demonstrated for MEQs that failed in over 50% to test more than mere recall of knowledge [15, 20]. Intensive and repeated training of panelists might be necessary.

Our study revealed that PR/diagnosis questions occurred in more than 70% of all identified PRQs that used only the first step of the clinical reasoning process [21] as their basic concept. In surgery and internal medicine, we detected the largest numbers of PR/therapy and PR/diagnostic procedures questions. These provide an additional step upwards in the taxonomy, because they include the interpretation of a pattern’s meaning and the application of additional knowledge [21]. Thereby, this type of PRQ includes not only typical signs or symptoms of a disease, but also additional information, such as certain laboratory results, as part of a pattern. According to the dual-process theory, additional information is usually obtained in the clinical reasoning process by active collection [22], which cannot be simulated in MCQs. A cognitive model resembling pattern recognition, including additional information, has recently been developed, in order to generate multiple-choice test items [23]. This could be very helpful in designing PRQs at a taxonomically higher order and, therefore, we suggest that pattern recognition should be added as a specific medical concept to MCQ item writing guidelines [24].

Using PRQs more frequently in MCQ exams to increase the cognitive level of the questions cannot be concluded from our study without additional considerations. A possible reason why PR/diagnosis questions occur mostly in neurology and pediatrics could be the high availability of disease patterns in these disciplines [25, 26]. This is especially true for the core neurological diagnostic approach of logically localizing a neural lesion, which translates well into the concept of pattern recognition. However, it must be noted that patterns in these disciplines often define rare diseases with greater relevance in postgraduate medical education within these specific disciplines. Therefore, the use of PRQs in exams for undergraduate medical students should preferably be in alignment with the content and specificity of the respective medical curriculum [27]. Furthermore, it has been demonstrated that the skill of pattern recognition as a non-analytical model of clinical reasoning increases with experience [28]. To train pattern recognition skills longitudinally and to provide an alignment of medical undergraduate training with PRQs in high stakes exams, students should have sufficient opportunities to practice the skill of pattern recognition and to receive supervision and feedback for their learning process [13, 22]. Another opportunity to teach diagnostic patterns could be the use of virtual patients [29] or electronically available PRQs and also IPRQs, where patterns can be highlighted in the learning process.

A limitation of our study is that it only included the four largest disciplines and excluded 19 smaller disciplines from the original analysis, albeit for statistical reasons. As another limitation, we only studied high stakes exams from one country. However, the framework we suggest for categorizing MCQs can be applied easily in all disciplines and countries and provides an additional concept for quality analysis of MCQ based exams. Furthermore, this framework provides a tool to design MCQs for applied knowledge using pattern recognition as the basis to test diagnostic and therapeutic strategies. It could also be helpful to find the desired balance between MCQs testing factual knowledge and applied knowledge while the amount of MCQs for applied knowledge might be higher in exams at the threshold of postgraduate medical education.

## Conclusions

Pattern recognition is a prominent concept in MCQs from a National Medical Licensing Exam to test the application of clinical knowledge. Panelists involved in designing questions for high stakes exams should be aware of the PR concept in MCQs, in order to create PRQs with different emphases, depending on the requirements of the individual discipline. Undergraduate medical students should be provided with longitudinal learning opportunities for clinical reasoning, including feedback on their pattern recognition skills in the application of their knowledge. The quality of questions for applied knowledge in MCQ-based exams can be increased by using questions with unambiguous medical patterns to assess the clinical reasoning processes.
